# Serum cytokines in second trimester pregnancy and their relationship with spontaneous preterm births in the Ribeirão Preto and São Luiz cohorts

**DOI:** 10.1186/s12884-023-05791-3

**Published:** 2023-06-21

**Authors:** Suzana Eggers Turra, Ênio Luis Damaso, Eduardo Carvalho de Arruda Veiga, Viviane Cunha Cardoso, Heloisa Bettiol, Ricardo Carvalho Cavalli

**Affiliations:** 1 Department of Gynecology and Obstetrics, Ribeirao Preto Medical School, University of Sao Paulo, Ribeirao Preto, Sao Paulo, Brazil; 2 Department of Pediatric Dentistry, Orthodontics and Collective Health, Faculty of Dentistry of Bauru, University of São Paulo, Bauru, SP Brazil; 3 Faculdade de Medicina de Ribeirão Preto, Universidade de São Paulo, Avenida: Bandeirantes, 3900, Sao Paulo, CEP: 14049-900 Brazil; 4 Department of Pediatrics, Ribeirao Preto Medical School, University of Sao Paulo, Ribeirao Preto, Sao Paulo, Brazil

**Keywords:** Cytokines, Preterm birth, Prenatal cohorts

## Abstract

**Objective:**

To evaluate the association between second trimester plasma cytokine levels in asymptomatic pregnant women and preterm births (PTB) in an attempt to identify a possible predictor of preterm birth.

**Methods:**

The study design was a nested case–control study including women with singleton a gestational age between 20–25(+ 6) weeks from two Brazilian cities. The patients were interviewed, Venous blood samples were collected. The participants were again evaluated at birth. A total of 197 women with PTB comprised the case group. The control group was selected among term births (426 patients). Forty-one cytokines were compared between groups.

**Results:**

When only spontaneous PTB were analyzed, GRO, sCD40L and MCP-1 levels were lower in the case group (*p* < 0.05). Logarithmic transformation was performed for cytokines with discrepant results, which showed increased levels of IL-2 in the group of spontaneous PTB (*p* < 0.05). In both analyses, the incidence of maternal smoking and of a history of preterm delivery differed significantly between the case and control groups. In multivariate analysis, only serum GRO levels differed between the case and control groups.

**Conclusion:**

Lower second trimester serum levels of GRO in asymptomatic women are associated with a larger number of PTB. This finding may reflect a deficient maternal inflammatory response.

## Introduction

Preterm birth (PTB) is defined as birth before 37 weeks of gestation, which may or may not be preceded by labor, and is independent of the newborn’s weight [1]. Its incidence varies widely between developed and developing countries and has shown a significant global increase in recent years [2]. Preterm births are responsible for about 75% of neonatal deaths and the surviving newborns are susceptible to numerous complications, including respiratory disorders, delayed neuropsychomotor development, cerebral palsy. and other neurological sequelae such as vision, hearing and motor deficits [3, 4].

The physiopathology of PTB is not fully understood but appears to be the result of a multifactorial process in which the interaction of numerous factors transforms the quiescent uterus into an effectively contracting uterus [5]. Preterm labor can be caused by activation of the normal labor process or be the consequence of risk conditions and pathological processes [6]. The isolated or simultaneous occurrence of some of these pathological processes will lead to a common pathway that is characterized by degradation of the extracellular matrix of the cervix and fetal membranes and by myometrial stimulation, accompanied by uterine contractions, cervical dilatation and membrane rupture [7]. Studies have suggested the involvement of some cytokines in the onset of labor and in the pathological processes [8].

Several studies have reported a strong association of high concentrations of inflammatory biomarkers with a short cervix [9] and consequently with PTB, which may be caused by increased levels of the cytokines that triggers rupture of the cervical connective tissue [10]. Interleukin 1 beta (IL-1β) and tumor necrosis factor alpha (TNF-α) stimulates the production of prostaglandins in the myometrium. Interleukin 6 (IL-6) stimulates the production of acute-phase proteins and enzymes necessary for prostaglandin production, while IL-8 is important for modification and preparation of the uterine cervix [11].

Cytokines are hydrosoluble glycoproteins or polypeptides that are synthesized and released by different cell types in the organism in response to different stimuli. They can be divided into interleukins (IL-1 to IL-35), chemotactic cytokines (chemokines), tumor necrosis factors, mesenchymal growth factors, and interferons [12]. During pregnancy, cytokines trigger contractions and are involved in the onset of labor, in addition to balancing the inflammatory response during the period. Cytokines are also responsible for the maintenance of pregnancy and for the fact that the fetus is not recognized as a foreign body by the maternal immune system [8].

The well-established relationship between inflammation and myometrial contraction that underlies the mechanism of contractions and subsequent progression to labor (preterm or term) allows us to question whether the early emergence of inflammatory markers in maternal venous blood an indicator of PTB may be and whether these markers may be used for population screening because of the easy application of this method and noninvasive sample collection. In view of this association, studies have attempted to determine the role of cytokines in the diagnosis of labor by measuring their levels in vaginal secretions, amniotic fluid, and/or maternal blood. However, these studies are conflicting since their results are variable and inconsistent [13].

The aim of this study was to establish the relationship between serum cytokine levels in asymptomatic women in the second trimester of gestation and PTB, and to identify cytokines that could be used as risk indicators of prematurity in asymptomatic patients in the second trimester.

## Methods

### Study design

The study design was a case–control study nested within a convenience cohort that is part of a project entitled “Etiological factors of preterm birth and consequences of perinatal factors in child health: birth cohorts in two Brazilian cities” (BRISA project). The BRISA project was approved by the Ethics Committee under number 4116/2008.

The participants were recruited through the municipal health system in Ribeirão Preto (SP) and São Luís (MA) between February 2010 and February 2011. The patients were invited to participate in the project during regular prenatal consultations and those who showed interest were asked to come to the hospitals responsible for data collection in their respective cities: University Hospital of the Ribeirão Preto Medical School (HCRP) in Ribeirão Preto, State of São Paulo, and three maternity units in São Luís, State of Maranhão, Brazil.

### Inclusion and exclusion criteria

Exclusion criteria were multiple pregnancies and fetuses with congenital malformations or chromosomal syndromes diagnosed before 20 weeks or at the time of prenatal data collection (confirmed with ultrasound). The inclusion criteria are only pregnant women with a single fetus and a gestational age between 20–25(+ 6) weeks (confirmed by obstetric ultrasound performed at less than 20 weeks of gestation) were included and term of consent and clarification of the study that the participant signed for anonymous use of the data collected. All patients who agreed to participate in the study signed the free informed consent form. Women with multiple pregnancies and fetuses with congenital malformations or chromosome syndromes diagnosed before 20 weeks or on the occasion of prenatal data collection (confirmed by ultrasound) were excluded.

### Sample collection

The pregnant women eligible for the study answered a standard questionnaire and underwent an interview and examinations, including gynecological examination, morphological and transvaginal obstetric ultrasound for the measurement of cervical length (except for pregnant women from São Luís where the uterine cervix is not measured routinely), and collection of a venous blood sample. The team responsible for the collection and processing of the data was adequately trained to ensure homogeneity and applicability of the assessment.

The study involved 2,864 pregnant women from Ribeirão Preto and São Luís. Based on the responses of the birth cohort in the BRISA project, it was possible to identify and exclude from the case.

**Fig. 1:**
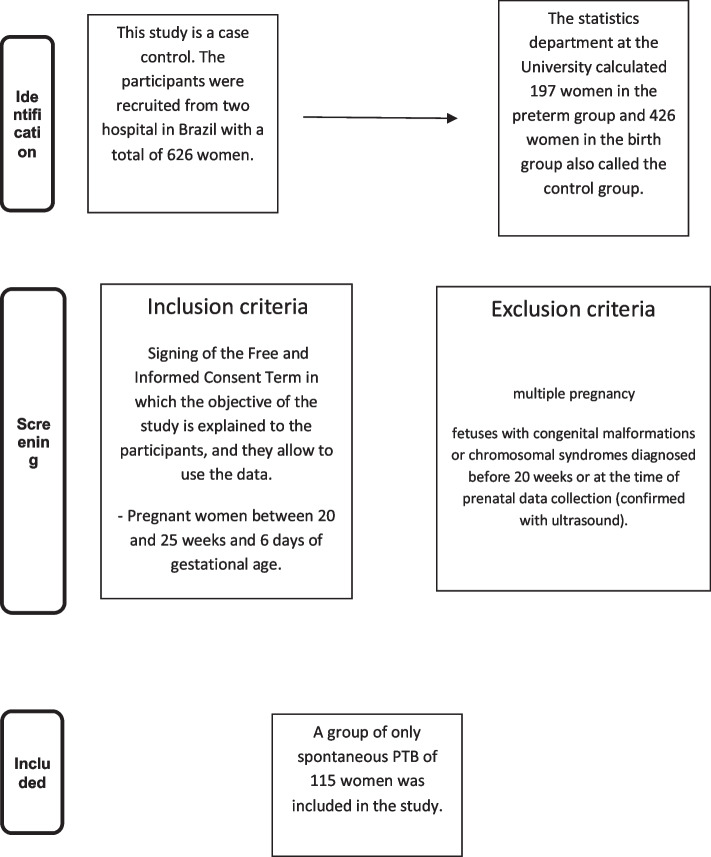
Flow chart of the study

Group patients who progressed to PTB because of obstetric indications (*n* = 82), constituting a new group (*n* = 115) of cases that contained only spontaneous PTB (spontaneous PTB group). Spontaneous PTB was defined as that resulting from preterm labor or chorioamniorrhexis. A premature (PTB) baby is a child who was born preterm, that is, before completing 37 weeks of gestation [14]. The control group (pregnant women who delivered at term, ≥ 37 weeks) was selected by simple random drawing without replacement from the remaining cohort at a proportion of 2:1 in their respective cities (426 patients). Thus, the total sample of the study consisted of 623 patients (Fig. 1).

For cytokine analysis, venous blood samples were collected at the time of assessment of the prenatal cohort and sent to the laboratory for centrifugation and separation of serum. The serum samples were stored in a freezer for subsequent analysis in the case and control groups. The serum inflammatory markers (IL-1ß, IL-6, IL-8, IL-10 and TNF-α, among other cytokines, totaling 41 different markers) were measured using a high-sensitivity assay (Milliplex Map Human Cytokine/Chemokine Panel, Cat HCYTOMAG-60 K-PX41, Millipore Corporation, Billerica, MA, USA) according to manufacturer recommendations. The analytes were quantified in a Luminex 200 analyzer (Millipore Corporation, Billerica, MA, USA) using the Analyst Software and the results are expressed as pg/ml.

The seven most studies cytokines associated with PTB were measured: TNF, IFN-γ, IL-1, IL-6, IL-17 (proinflammatory), and TGF-ß and IL-10 (anti- inflammatory), added to the 41 markers available in the commercial kit used in the study.

Variables that could influence the number of PTB were also analyzed [15]: maternal age (< 20, 20 to 34, ≥ 35 years), parity (1, 2–3, ≥ 4 births), history of PTB (yes and no), smoking (yes, if the patient had smoked at least one cigarette per day during pregnancy, and no), and genitourinary infection (urinary tract infection confirmed by urine culture and/or bacterial vaginosis confirmed by clinical and microscopic examination). Cervical length was not considered since this parameter was not evaluated in patients from São Luís. The following data were collected at birth: city and hospital of birth, date of birth, and gestational age at birth in days.

### Statistical analysis

Statistical analysis was performed in two steps. First, all women with PTB that had their cytokines measured in the prenatal cohort of the BRISA project were included and comprised the case group (PTB group). The following variables were compared between the case and control groups: maternal age, parity, history of PTB, smoking and presence of genitourinary infection, as well as the 41 inflammatory markers. Second, based on the responses of the birth cohort in the BRISA project, it was possible to identify and exclude from the case group patients who progressed to PTB because of obstetric indications (*n* = 82), constituting a new group (*n* = 115) of cases that contained only spontaneous PTB (spontaneous PTB group). Spontaneous PTB was defined as that resulting from preterm labor or chorioamniorrhexis.

The data were tabulated in an Excel spreadsheet and then exported to the SAS 9.3 program (SAS Institute, Inc., 2010). First, exploratory analysis was performed using measures of central tendency and dispersion and box plots. The Student t-test was used to compare mean values of the variables of interest between the two groups. The level of significance was set at 5%. The proc t test procedure of the software was used for all tests. For variables showing wide variability whose distribution was not symmetrical, logarithmic transformation was performed and the results were compared to those obtained with the tests without transformation to evaluate the influence of outliers. Variables that differed between the case and control groups were then submitted to multivariate analysis.

## Results

A total of 2,864 pregnant women from Ribeirão Preto and São Luís were included in the study. There were 242 women with PTB (8.7% of all patients, *n* = 2,757). Forty-five PTB were excluded from the study because of insufficient data or the impossibility of analyzing inflammatory markers (not collected, inadequate sample). Spontaneous PTB group was *n* = 115 of cases. The control group (pregnant women who delivered at term, ≥ 37 weeks) was selected by simple random drawing without replacement at a proportion of 2:1 in their respective cities (426 patients). Thus, the total sample of the study consisted of 623 patients. In the group that included only spontaneous PTB (spontaneous PTB group), the mean age at birth was 239.8 days.

Exclusive comparison between the spontaneous PTB group (*n* = 115) and the control group revealed no expressive changes in relation to those observed in the PTB group as a whole. A significant difference was only observed for maternal smoking and a history of preterm delivery in previous pregnancies. Maternal age, parity or infections did not differ between the two groups (Table 1).

**Table 1:** Clinical characteristics of women with spontaneous preterm birth (spontaneous PTB group) and women who delivered at term (control group). BRISA Cohort, Ribeirão Preto and São Luís, 2010

Variables	Spontaneous PTB group		Control group		*P*^1^
	**n**	**%**	**n**	**%**	
**Maternal age (years)**					
≤ 20	11	9.6	35	8.2	0.5362
20–34	95	82.7	368	86.4	
≥ 35	9	7.7	23	5.4	
Total	115	100	426	Total	
**Parity**					
1	51	44.3	200	46.9	0.8412
2—3	51	44.3	184	43.2	
≥ 4	13	11.4	42	9.9	
Total	115	100	426	100	
**Smoking**					
Yes	26	22.6	63	14.8	0.0447
No	89	77.4	363	85.2	
Total	115	100	426	100	
**Previous preterm delivery**					
Yes (1 or more)	74	64.3	29	6.8	≤ 0.0001
No	41	35.7	397	93.2	
Total	115	100	426	100	
**Genitourinary infections**^**a**^					
Yes	38	33.1	102	23.9	0.0653
No	77	66.9	324	76.1	
Total	115	100	426	100	

^1^Chi-squared test

^a^Urinary tract infection confirmed by urine culture and/or bacterial vaginosis confirmed by clinical and microscopic examination

A significant difference between the two groups was also observed for cytokines GRO, with lower levels in patients with spontaneous PTB. On the other hand, this analysis revealed a significant difference in MCP-1 (monocyte chemoattractant protein 1), whose levels were also lower in the spontaneous PTB group (Table 2).

**Table 2:** Comparison of the mean levels and standard deviation of 41 cytokines between women with spontaneous preterm birth (PTB group) and women who delivered at term (control group). BRISA Cohort, Ribeirão Preto and São Luís, 2010

Cytokine	Group	Mean (pg/ml)	SD	*p*	Cytokine	Group	Mean (pg/ml)	SD	*p*
EGF	Spontaneous PTB	176.5	121.8	0.70	Log_IL-17A	Spontaneous PTB	34.7	58.5	0.95
	Control	171.7	122.5			Control	41.6	88.0	
Log_FGF-2	Spontaneous PTB	141.4	84.4	0.54	Log_IL-1Ra	Spontaneous PTB	104.7	103	0.54
	Control	138	91.4			Control	114	237.5	
Log_Eotaxin	Spontaneous PTB	110.4	77.0	0.30	Log_IL-1 alpha	Spontaneous PTB	53.5	57.5	0.61
	Control	117.2	86.1			Control	55.8	83.1	
Log_TGF-alpha	Spontaneous PTB	17.5	22.0	0.16	Log_IL-9	Spontaneous PTB	5.1	5.4	0.18
	Control	21.3	27.2			Control	7.8	32.9	
G-CSF	Spontaneous PTB	153.4	106.5	0.63	Log_IL-1 beta	Spontaneous PTB	5.5	5.0	0.12
	Control	159.8	131			Control	5.8	11.7	
Flt-3L	Spontaneous PTB	59.6	62.4	0.34	Log_IL-2	Spontaneous PTB	15.5	40.3	0.03
	Control	69.3	103.7			Control	11.0	31.7	
Log_GM-CSF	Spontaneous PTB	64.3	76.1	0.23	Log_IL-3	Spontaneous PTB	3.5	3.1	0.02
	Control	60.3	80.1			Control	3.1	4.9	
Log_Fractalkine	Spontaneous PTB	87.7	60.4	0.12	Log_IL-4	Spontaneous PTB	52.2	92.5	0.14
	Control	88.7	79.2			Control	45.4	89.0	
IFN-A2	Spontaneous PTB	79.2	53.3	0.80	Log_IL-5	Spontaneous PTB	4.4	7.2	0.12
	Control	77.7	60.9			Control	4.9	11.4	
Log_IFN-gamma	Spontaneous PTB	55.5	87.3	0.47	Log_IL-6	Spontaneous PTB	20.3	24.0	0.10
	Control	64.1	136.4			Control	19.8	45.1	
Log_GRO	Spontaneous PTB	1560.1	809.5	0.006	IL-7	Spontaneous PTB	37.8	48.6	0.86
	Control	1950.6	1295.9			Control	38.8	57.6	
Log_IL-10	Spontaneous PTB	9.9	10.6	0.56	Log_IL-8	Spontaneous PTB	59.9	94.6	0.46
	Control	14.1	51.2			Control	64.1	95.4	
Log_MCP-3	Spontaneous PTB	58.2	69.1	0.03	IP10	Spontaneous PTB	413.4	245.5	0.54
	Control	53.9	69.9			Control	428.9	238.3	
Log_IL-12P40	Spontaneous PTB	48.2	47.7	0.21	MCP-1	Spontaneous PTB	377.5	206.7	0.01
	Control	42.2	46.4			Control	433.1	224.2	
MDC	Spontaneous PTB	1628.3	790.7	0.24	Log_MIP-1A	Spontaneous PTB	35.6	99.1	0.57
	Control	1745.7	1005.3			Control	76.0	517.3	
IL-12P70	Spontaneous PTB	28.9	62.8	0.53	MIP-1B	Spontaneous PTB	86.1	85.92	0.76
	Control	49.5	28.9			Control	83.4	86.9	
Log_PDGF-AA	Spontaneous PTB	50.6	51.4	0.01	RANTES	Spontaneous PTB	10,062.5	5834.2	0.31
	Control	10.7	69.6			Control	10,784.8	7044.1	
Log_IL-13	Spontaneous PTB	14.9	18.8	0.16	Log_TNF-alpha	Spontaneous PTB	16.3	24.7	0.47
	Control	14.8	24.9			Control	17.0	21.2	
PDGF-BB	Spontaneous PTB	27,225.8	12,172.9	0.16	Log_TNF-beta	Spontaneous PTB	26.2	36.9	0.49
	Control	28,924.3	11,311.4			Control	29.5	69.1	
Log_IL-15	Spontaneous PTB	17.2	48.6	0.15	Log_VEGF	Spontaneous PTB	319.1	272.3	0.54
	Control	13.3	38.5			Control	361.8	646.6	
SCD40L	Spontaneous PTB	34.3	28.5	0.008					
	Control	42.6	30.1						

Cytokines: *EGF* Epidermal growth factor, *FGF2* Fibroblast growth factor, *TGF-alpha* Transforming growth factor alpha, *G-CSF* Granulocyte colony-stimulating factor, *Flt-3L* Fms-related tyrosine kinase 3, *GM-CSF* Granulocyte macrophage colony stimulating factor, *IFN-A2* Interferon alpha 2, *IFN-gamma* Interferon gamma, *GRO* Growth related oncogene, *IL-10* Interleukin 10, *MCP-3* Monocyte chemoattractant protein 3, *IL12p40* Interleukin 12p40, *MDC* Monocyte chemotactic protein, *IL12p70* Interleukin 12p70, *PDGF-AA* Platelet-derived growth factor AA, *IL-13* Interleukin 13, *PDGF-BB* Platelet-derived growth factor BB, *IL-15* Interleukin 15, *SCD40L* Soluble CD40-ligand, *IP10* Interferon gamma-induced protein 10, *MCP1* Monocyte chemoattractant protein 1, *MIP* Macrophage inflammatory protein, *TNF* Tumor necrosis factor, *VEFG* Vascular endothelial growth factor, *RANTES* Regulated on activation, normal T cell expressed and secreted

Logarithmic transformation was performed for cytokines with very discrepant values from the mean (outliers) and the results were compared with those obtained by tests without transformation to identify the possible influence of these outliers. Among the reevaluated cytokines, a significant difference was observed for Log IL-2 in the group involving only spontaneous PTB (*p* = 0.03) (Table 2).

In multivariate analysis, only a history of preterm delivery and GRO were associated with PTB. Patients with a history of preterm delivery had an almost 7 times higher risk of PTB (RR: 7.63; 95%CI: 5.20, – 11.23). As the measurement unit of GRO increased, the risk of PTB decreased. Since the marker-related variables are continuous, the estimates were very close to 1 (Table 3).

**Table 3:** Unadjusted and adjusted relative risk, 95% confidence interval and *p*-value involving clinical characteristics and inflammatory factors

Variable	Unadjusted RR	95% CI		*p*	Adjusted RR	95% CI		*p*
		**LL**	**UL**			**LL**	**UL**	
Previous preterm delivery	7.63	5.20	11.23	< .0001	7.25	4.91	10.70	< .0001
Smoking pregnant	1.48	0.95	2.29	0.077	1.25	0.799	1.95	0.3271
GRO	1.00	1.00	1.00	0.006	0.99	0.99	1.00	0.0344
IL-2	1.00	1.00	1.01	0.269	1.00	0.99	1.00	0.5551
SCD40L	1.00	1.00	1.00	0.019	1.00	1.00	1.00	0.0649
MCP-1	1.01	1.00	1.01	0.034	0.99	0.99	1.00	0.1619
IL-3	1.00	0,98	1.03	0.495	0.99	0.93	1.06	0.9659
MCP-3	1.00	0,99	1.00	0.606	0.99	0.99	1.00	0.8693
PDGF-AA	1.00	1.00	1.00	0.478	1.00	1.00	1.00	0.8297

*RR* Relative risk, *95% CI* 95% confidence interval, *LL* Lower limit, *UL* Upper limit

## Discussion

The present study evaluated characteristics throughout the prenatal period that could be associated with PTB, more specifically spontaneous PTB, i.e., that resulting from preterm labor or rupture of the chorioamniotic membranes. For this purpose, maternal characteristics and serum cytokine concentrations were analyzed in two groups of patients for subsequent calculation of the relative risk in univariate and multivariate analyses.

Studies have investigated risk factors of prematurity and the most important maternal risk factors include extreme ages [15, 16], smoking [15, 17, 18], genitourinary infections [19], and a history of preterm delivery [15, 20]. The last is the main risk factor associated with prematurity; the larger the number of previous PTB, the greater the risk of prematurity [15, 21].

Our results only identified a history of at least one preterm delivery as a risk factor for spontaneous PTB, while no association was observed with maternal age, parity, smoking or genitourinary infection. These discrepant findings might be related to the small sample size, the choice of the control group, and the fact that a convenience cohort was studied.

It is important to characterize that PTB has an increase in inflammatory mediators such as IL-1, IL-6, IL-8, TNFα, as well as a decrease in other inflammatory mediators such as IL-10 and IL-4, and this balance is essential to maintain delivery with normal weight or PTB [22]. In this sense, the cytokines studied in this study had their evaluations and among those that were significant are the cytokines GRO and MCP1. In the study by Sullivan et al. 2002, the cutoff value for serum cytokine in preterm infants was defined as around 250 pg/ml. In another study from 2021 but on the serum cytokine MCP1 the cut-off values of normal children are around 150 ng/ml and in this work several cytokines were compared with children with sepsis [23] it is also worth remembering that we are talking about very specific cytokines that there are not many articles in the literature, especially when it comes to spontaneous preterm births. Immune modulation systematically expressed throughout a pregnancy result in less complicated deliveries than spontaneous preterm deliveries, including data from women who smoke confirm this trend of complications in childbirth [24, 25].

The use of antibiotics during pregnancy can be a treatment for bacterial infections, but there are indications that these patients with the use of antibiotics may be more likely to have direct or indirect preterm delivery and also inflammatory reactions [26].

In addition to maternal characteristics, we examined the serum concentrations of inflammatory markers (cytokines, chemokines, and growth factors) in asymptomatic pregnant women in the second trimester and compared them between patients with PTB and a group of patients who delivered at term. Among the 41 cytokines analyzed, only GRO was a risk factor for spontaneous prematurity. The mean level of this cytokine was lower in the PTB group, although the literature shows an association between increased serum proinflammatory cytokines and PTB [27].

GRO is a chemokine that consists of three subunits: GROα/CXCL1, GROβ/CXCL2, and GROγ/CXCL3. This chemokine is produced by different cell types such as synovial cells, monocytes, fibroblasts, and endothelial cells. Its production is directly influenced by 7 IL-8, which is responsible for neutrophil activation and chemotaxis of inflammatory cells [28]. Some studies have shown that an increase in chemokines [29] of the CXC family in maternal serum [30] and in amniotic fluid of patients with chorioamnionitis [29] was associated with PTB.

Our findings are biologically plausible. Laudanski et al. [31] also found significantly lower serum GRO concentrations in pregnant women with preterm labor compared to women who delivered at term (> 37 weeks of gestation), but did not establish a causal relationship between the serum concentration of this cytokine and progression to prematurity. On the other hand, Hsu et al. [32] detected higher concentrations of the alpha subunit of this cytokine in patients who progressed to PTB. Low serum GRO levels may reflect a deficient inflammatory response and increased susceptibility to infections, increasing the risk of triggering a myometrial contractile response and PTB. Some studies have demonstrated that lower serum or cervical concentrations of proinflammatory cytokines can predispose to infection and chorioamnionitis, which can cause PTB [33], in agreement with the findings of the present study.

The analysis of this study may be compromised by the fact that it was not possible to assess changes in the plasma concentrations of the inflammatory markers throughout pregnancy, which would allow to identify critical periods during which cytokine levels would reflect the real increase in the risk of prematurity. Another limitation of the study is the impossibility of evaluating cervical characteristics and their association with serum levels of the inflammatory markers since the patients from São Luís did not undergo specific ultrasound examination to measure cervical length in the prenatal cohort, and another limitation is the use of corticosteroids during pregnancy, which can cause lung or neurological damage in the neonate, future randomized clinical trials will bring more evidence for this indication. Normal cut-off values for the cytokines are not available in the literature for direct comparison with the outcome (prematurity). The results of different studies are still insufficient to indicate the serum concentration of inflammatory markers in asymptomatic patients in the second trimester as a parameter to predict PTB or to identify at-risk populations.

## Data Availability

The datasets used and/or analyzed during the current study available from the corresponding author on reasonable request.

## References

[1] ACOG Committee Opinion No 579: Definition of term pregnancy. Obstet Gynecol. 2013;122(5):1139-40. 10.1097/01.AOG.0000437385.88715.4a. 10.1097/01.AOG.0000437385.88715.4a 24150030

[2] Wang H, Naghavi M, Allen C, Barber RM, Bhutta ZA, Carter A (2016). Global, regional, and national life expectancy, all-cause mortality, and cause-specific mortality for 249 causes of death, 1980–2015: a systematic analysis for the Global Burden of Disease Study 2015. Lancet.

[3] Kim JK, Chang YS, Sung S, Park WS (2019). Mortality rate-dependent variations in the survival without major morbidities rate of extremely preterm infants. Sci Rep.

[4] Chattopadhyay N, Mitra K. Neurodevelopmental outcome of high risk newborns discharged from special care baby units in a rural district in India. J Public Health Res. 2015;4(1):318. 10.4081/jphr.2015.318. 10.4081/jphr.2015.318 PMC4407034 25918689

[5] World Health Organization, March of Dimes, Partnership for Maternal, Newborn and Child Health, Save the Children. Born too soon: the global action report on preterm birth, 2012. Available: https://www.who.int/pmnch/media/news/2012/201204_borntoosoon-report.pdf.

[6] Goldenberg RL, Culhane JF, Iams JD, Romero R (2008). Epidemiology and causes of preterm birth. Lancet Lond Engl.

[7] Lockwood CJ, Kuczynski E (1999). Markers of risk for preterm delivery. J Perinat Med.

[8] Yockey LJ, Iwasaki A (2018). Interferons and proinflammatory cytokines in pregnancy and fetal development. Immunity.

[9] Vogel I, Goepfert AR, Thorsen P, Skogstrand K, Hougaard DM, Curry AH (2007). Early second-trimester inflammatory markers and short cervical length and the risk of recurrent preterm birth. J Reprod Immunol outubro de.

[10] Sennström MKB, Brauner A, Lu Y, Granström LMM, Malmström AL, Ekman GE (1997). Interleukin-8 is a mediator of the final cervical ripening in humans. Eur J Obstet Gynecol Reprod Biol.

[11] Blackburn S (2008). Cytokines in the perinatal and neonatal periods: selected aspects. J Perinat Neonatal Nurs.

[12] de Oliveira CMB, Sakata RK, Issy AM, Gerola LR, Salomão R (2011). Cytokines and pain. Braz J Anesthesiol.

[13] Ruiz RJ, Jallo N, Murphey C, Marti CN, Godbold E, Pickler RH (2012). Second trimester maternal plasma levels of cytokines IL-1Ra, Il-6 and IL-10 and preterm birth. J Perinatol.

[14] Dias BAS, Leal MC, Martinelli KG, Nakamura-Pereira M, Estevas-Pereira AP, Neto ETS (2022). Recurrent preterm birth: data from the study “Birth in Brazil”. Saúde Publica.

[15] Damaso EL, Rolnik DL, de Cavalli RC, Quintana SM, Duarte G, da Silva Costa F (2019). Prediction of preterm birth by maternal characteristics and medical history in the Brazilian population. J Pregnancy..

[16] Fuchs F, Monet B, Ducruet T, Chaillet N, Audibert F (2018). Effect of maternal age on the risk of preterm birth: a large cohort study. PLoS One.

[17] Kyrklund-Blomberg NB, Cnattingius S (1998). Preterm birth and maternal smoking: risks related to gestational age and onset of delivery. Am J Obstet Gynecol.

[18] Cui H, Gong T-T, Liu C-X, Wu Q-J (2016). Associations between passive maternal smoking during pregnancy and preterm birth: evidence from a meta- analysis of observational studies. PLoS One.

[19] Hillier SL, Nugent RP, Eschenbach DA, Krohn MA, Gibbs RS, Martin DH (1995). Association between bacterial vaginosis and preterm delivery of a low- birth-weight infant. The Vaginal Infections and Prematurity Study Group. N Engl J Med.

[20] Esplin MS, O’Brien E, Fraser A, Kerber RA, Clark E, Simonsen SE (2008). Estimating recurrence of spontaneous preterm delivery. Obstet Gynecol.

[21] Laughon SK, Albert PS, Leishear K, Mendola P (2014). The NICHD consecutive pregnancies study: recurrent preterm delivery by subtype. Am J Obstet Gynecol.

[22] Areia AL, Mota-Pinto A (2022). Inflammation and preterm birth: a systematic review. Reprod Med.

[23] Hassuna NA, Elgezawy E, Mousa SO, AbdelAziz RA, Ibrahem RA, Wahed WYA (2021). Diagnostic value of monocyte chemoattractant Protein-1, soluble mannose receptor, Presepsin, and Procalcitonin in critically ill children admitted with suspected sepsis. BMC Pediatr.

[24] Denney JM, Nelson EL, Wadhwa PD, Waters TP, Mathew L, Chung EK (2011). Longitudinal modulation of immune system cytokine profile during pregnancy. Cytokine.

[25] Denney JM, Nelson E, Wadhwa P, Waters T, Mathew L, Goldenberg RL (2021). Cytokine profiling: variation in immune modulation with preterm birth vs. Uncomplicated term birth identifies pivotal signals in pathogenesis of preterm birth. J Perinat Med.

[26] Cantarutti A, Rea F, Franchi M, Beccalli B, Locatelli A, Corrao G (2021). Use of antibiotic treatment in pregnancy and the risk of several neonatal outcomes: a population-based study. Int J Environ Res Public Health.

[27] Lyon D, Cheng C-Y, Howland L, Rattican D, Jallo N, Pickler R (2010). Integrated review of cytokines in maternal, cord, and newborn blood: part I–associations with preterm birth. Biol Res Nurs.

[28] Geiser T, Dewald B, Ehrengruber MU, Clark-Lewis I, Baggiolini M (1993). The interleukin-8-related chemotactic cytokines GRO alpha, GRO beta, and GRO gamma activate human neutrophil and basophil leukocytes. J Biol Chem.

[29] Aminzadeh F, Ghorashi Z, Nabati S, Ghasemshirazi M, Arababadi MK, Shamsizadeh A (2012). Differential expression of CXC chemokines CXCL10 and CXCL12 in term and pre-term neonates and their mothers. Am J Reprod Immunol.

[30] Hsu C-D, Meaddough E, Aversa K, Copel JA (1998). The role of amniotic fluid L- selectin, GRO-α, and interleukin-8 in the pathogenesis of intraamniotic infection. Am J Obstet Gynecol.

[31] Laudanski P, Lemancewicz A, Kuc P, Charkiewicz K, Ramotowska B, Kretowska M (2014). Chemokines profiling of patients with preterm birth. Mediators Inflamm.

[32] Weissenbacher T, Laubender RP, Witkin SS, Gingelmaier A, Schiessl B, Kainer F (2013). Diagnostic biomarkers of pro-inflammatory immune- mediated preterm birth. Arch Gynecol Obstet.

[33] Tantengco OAG, Menon R. Breaking Down the Barrier: The Role of Cervical Infection and Inflammation in Preterm Birth. Front Glob Womens Health. 2022;2:777643. 10.3389/fgwh.2021.777643. 10.3389/fgwh.2021.777643 PMC8803751 35118439

